# Optimization of a Sports Activity Development Model Using Artificial Intelligence under New Curriculum Reform

**DOI:** 10.3390/ijerph18179049

**Published:** 2021-08-27

**Authors:** Taofeng Liu, Dominika Wilczyńska, Mariusz Lipowski, Zijian Zhao

**Affiliations:** 1 School of Physical Education Institute (Main Campus), Zhengzhou University, No. 100 Science Avenue, Zhengzhou 450001, China; 202033084@sangmyung.kr; 2 Department of Physical Education, Sangmyung University, Seoul 390-711, Korea; 3 Faculty of Physical Culture, Gdansk University of Physical Education and Sport, Kazimierza Górskiego 1, 80-336 Gdańsk, Poland; dominika.wilczynska@awf.gda.pl

**Keywords:** new curriculum reform, sports development mode, artificial intelligence, intelligent wearable devices, human action recognition

## Abstract

The recent curriculum reform in China puts forward higher requirements for the development of physical education. In order to further improve students’ physical quality and motor skills, the traditional model was improved to address the lack of accuracy in motion recognition and detection of physical condition so as to assist teachers to improve students’ physical quality. First, the physical education teaching activities required by the new curriculum reform were studied with regard to the actual needs of China’s current social, political, and economic development; next, the application of artificial intelligence technology to physical education teaching activities was proposed; and finally, deep learning technology was studied and a human movement recognition model based on a long short-term memory (LSTM) neural network was established to identify the movement state of students in physical education teaching activities. The designed model includes three components: data acquisition, data calculation, and data visualization. The functions of each layer were introduced; then, the intelligent wearable system was adopted to detect the status of students and a feedback system was established to assist teaching; and finally, the dataset was constructed to train and test the designed model. The experimental results demonstrate that the recognition accuracy and loss value of the training model meet the practical requirements; in the algorithm test, the motion recognition accuracy of the designed model for different subjects was greater than 97.5%. Compared with the traditional human motion recognition algorithm, the designed model had a better recognition effect. Hence, the designed model can meet the actual needs of physical education. This exploration provides a new perspective for promoting the intelligent development of physical education.

## 1. Introduction

A new understanding of the importance of education due to the rapid development of China’s social, political, and economic landscape has led to constant changes in the public’s requirements for school education [1]. As part of this education, physical education plays a crucial role; schools need to focus on cultivating students’ outlooks on life and social values, while also ensuring that students participate in physical activities and improve their physical quality [2,3]. Therefore, under the requirements of the new curriculum reform, physical education teaching activities need to be further managed and optimized to improve students’ physical quality and sports skills [4]. Wearable devices employing artificial intelligence benefit from portability, interactivity, pertinence, and safety, which together allow them to: identify the status of students in sports activities; improve the efficiency of physical education teaching; facilitate communication between students and teachers; realize intelligent, data-based, and visual research of sports teaching; and effectively enhance the quality of physical education teaching activities [5]. As the new curriculum reform sets high requirements for the improvement of students’ physical quality, it is essential to formulate student exercise plans that: teach students according to their aptitude, avoid exercises which are ineffective for individual students, and prevent the phenomenon of sudden death during exercise that has gradually increased over recent years. Wearable devices combined with machine vision technology have been adopted to create systems capable of recognizing and analyzing the physical condition of the user.

Wearable sensor systems are used to monitor the body state of athletes and are studied by scholars in China and numerous other countries. Tang (2021) studied the sports training management model of an embedded wearable device combined with machine vision technology, and demonstrated improvements to visual measurement, response, and execution in sport based on visual preparation behavior [6]. Wu et al. (2018) applied information and communication technology to an education curriculum to improve the efficacy of traditional teaching methods and ability training [7]. Wang and Park (2021) proposed the use of artificial intelligence technology to design an intelligent sports management system to address the problems of poor physical fitness in students and inefficient management of sports venues. A distributed microservice system was established wherein the student situation is analyzed using an artificial intelligence recommendation algorithm and appropriate sports are recommended. A recommended curriculum is then established according to the student’s preferences and other relevant factors. The experimental results demonstrate that physical education teaching systems based on artificial intelligence and deep learning can adapt to different requirements and improve the physical quality of students [8]. Seshadri et al. (2021) studied the application of wearable sensors in real-time monitoring of athlete body parameters, and used an artificial intelligence model and machine learning to mine the sensor data set so as to provide suitable training plans for different athletes [9]. Du (2021) applied wearable devices based on the Internet of things to the tracking of basketball shooting performance. In the training process, microsensors were employed to identify the state of players to better understand differences among basketball players [10].

The current research reveals that the use of wearable devices and other sensors can collect movement data. Artificial intelligence was combined with deep learning technology to identify and analyze the physical condition of students and athletes, and to improve teaching outcomes in physical education and training. There are currently few studies on the physical education teaching model required under the new curriculum reform. Hence, wearable devices using artificial intelligence technology are proposed for student physical education. Wearable devices are adopted to monitor the movement status and body data of students in various sports so as to achieve the purposes of continuous testing, helping teachers personalize teaching for students with different physical qualities, and building a student-centered sports mode. This innovative research uses an LSTM (long short-term memory) recurrent neural network (RNN) to process and analyze data from intelligent wearable devices in real-time, which can provide teachers with timely feedback on student performance in the process of physical education teaching to help them formulate corresponding training strategies effectively and to realize personalized teaching of students.

## 2. Application of Artificial Intelligence Wearable Devices in Physical Education under New Curriculum Reform

### 2.1. The Development Requirements of Physical Education Activities under the Background of New Curriculum Reform

Since the founding of new China, China attaches great importance to the development of physical education, and sets basic objectives for physical education to clarify the requirements of national sports development [11]. In 1952, China began to establish physical education colleges so as to cultivate high-quality physical education teachers with systematic physical education knowledge for national primary and secondary schools [12]. In 1953, Mao Zedong put forward the “three good” requirement, which was quickly introduced into education policy [13]. The requirement of “three good” corrected the national problem of neglected physical education, and raised Chinese student health to new historical heights. The development of science and technology posed new challenges to China’s educational concepts and policies with increasingly fierce competition in the international environment [14]. In 1999, China carried out the eighth basic curriculum reform and decided to comprehensively extend the reform of basic education, which was formally implemented in 2000.

In 2001, the experimental physical education curriculum standard was incorporated into the education reform experiment, and the traditional physical education classroom was reformed using advanced educational concepts and policies which created a massive number of creative physical education teaching cases. By 2011, after ten years of practical testing, the new physical education curriculum standard had achieved good results through the reform process, and its problems had also been exposed [15]. Therefore, the ministry of education revised the physical education curriculum standard, put forward the teaching idea of “student development at the center” with the purpose of “encouraging students”, and proposed that students should be better integrated into the physical education class. The importance of physical education teachers was also emphasized in the new curriculum standard, which required teachers to have advanced knowledge and teaching ideas, thereby opening up new curriculum standards for physical education and health [16]. By 2021, after years of development, the implementation of the new curriculum reform had promoted the development of Chinese physical education and solved the problems identified before the curriculum reform. However, new problems regarding the curriculum objectives, physical education organization, and teaching content selection were also exposed.

Based on the optimization of physical education teaching modes under the new curriculum standard, it was proposed to introduce intelligent wearable devices into physical education teaching activities and to use artificial intelligence technology to monitor and analyze the learning process of students’ sports skills. According to the requirements of different teaching contexts and the physical quality of students, student sports ability was explored to realize personalized physical education. In the teaching process, it is essential to highlight the central position of students, to give full play to the subjective initiative of students in physical education, to improve student physical quality and sports skills, and to provide them the happiness of sports in learning.

### 2.2. Application of Artificial Intelligence Wearable Devices in Physical Education Activities

**Figure 1:**
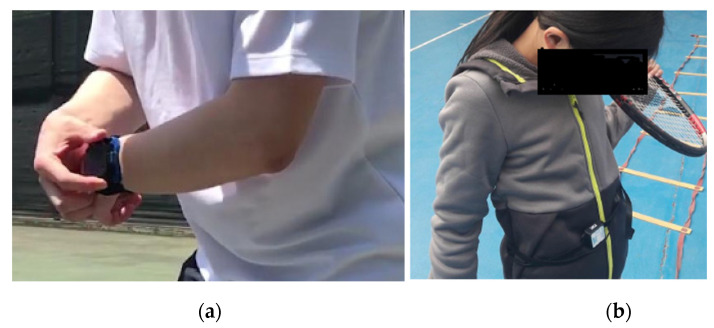
Using intelligent wearable devices to monitor the movement of students: (**a**) wearable motion recognition device for students, and (**b**) artificial intelligence wearable device for children.

The development of the Internet and information technology led to changes in data transmission and analysis. Under the background of the new curriculum reform, the education industry also needs to adapt to the development of the Internet and the social economy, align physical education with the development requirements of the times, construct teaching systems for quality physical education, and cultivate students who meet these needs of the times [17]. Artificial intelligence technology is based on computer science, cybernetics, and systems science. It advocates the use of knowledge to discover and solve problems that computers alone cannot handle, and has become the basis of human understanding and the transformation of the objective world in the information age [18]. With the demands of education curricula and the development of science and technology, artificial intelligence technology has become a crucial auxiliary means in both physical education and sports training, see Figure 1.

The introduction of artificial intelligence technology into physical education curriculum is an inevitability. At present, the health level of students in China reveals a downward trend. Strengthening the effect of physical exercise and testing and scientifically guiding student exercise methods are crucial components of physical education teaching reform under the new curriculum. Table 1 presents the main role of artificial intelligence wearable devices in sports activities.

### 2.3. Human Motion Recognition Algorithm Based on LSTM RNN

**Figure 2:**
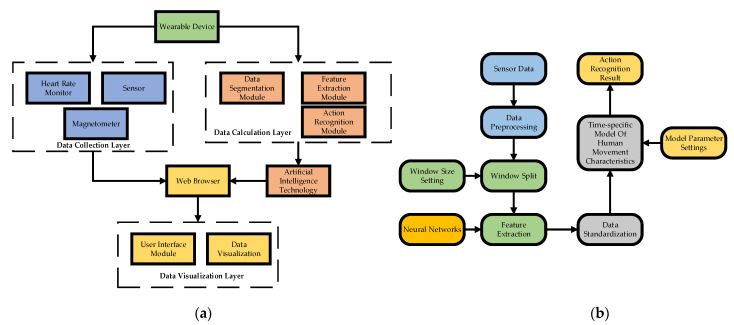
Human action recognition process based on wearable devices: (**a**) human action recognition system architecture, and (**b**) data-processing flow.

The popularity of wearable devices makes it more and more convenient to obtain human motion data. However, massive amounts of motion data need to be deeply mined to reflect the value of wearable devices [21]. The intelligence and informatization of physical education should be effectively improved to ensure that the data collected by intelligent wearable devices better serves the physical education course. It was proposed to analyze the data collected by intelligent wearable devices based on LSTM RNN, and to recognize the human movement of students in real-time [22]. LSTM can process the related data with a long interval before and after processing, and can automatically learn the features of data. Moreover, it can solve problems that traditional motion recognition classification algorithms ignore—such as the relevance of data features—and can improve the structure simplification and recognition efficiency of human action recognition pattern algorithms [23]. In the design of the human motion recognition algorithm, the algorithm considers three aspects: data acquisition, data calculation, and data visualization [24]. The data acquisition layer primarily collects and preprocesses the students’ movement data; the data calculation layer carries on the processes of storage, feature extraction, and motion recognition of motion data; and the data visualization layer displays the results of human motion recognition and real-time motion data through visualization technology [25]. Figure 2 presents the design of a human motion recognition system architecture based on wearable devices, as well as the process and identification flow of motion data.

#### 2.3.1. Data Acquisition Layer

The sensors commonly used in wearable devices include a three-axis gyroscope, a three-axis accelerometer, and a three-axis magnetometer, which are employed to collect the acceleration, angular velocity, and object orientation data of the tested object. However, sensors can experience noise problems during data measurement, including system noise and external vibration interference; therefore, the data needs to be processed. Common noise-processing methods include median filtering, Gaussian filtering, and Kalman filtering. However, filters with appropriate filtering effects are typically selected for different noise classes [26]. Besides noise, the problems of sensor sampling frequency and the time required for information acquisition need to be solved. Regarding missing data, the methods of deletion, mean interpolation, Lagrange interpolation, and median interpolation are adopted. The correlation among the inserted data can be ensured by mean interpolation. The missing values are estimated using the values before and after the missing data [27]. The data collected will fluctuate greatly when the range of human motion is large. Normalization or standardization is adopted to unify different dimensions of data, so as to avoid the effect of extreme data and the model recognition effect.

The normalization process is to compress a large range of data into the range of [0, 1]. The equation used is:

(1) $$x^{\prime }=\frac{x-x_{\min}}{x_{\max}-x_{\min}}$$

The standardization process is to process the large and small isoutlier data in the dataset, and transform them into the standard normal distribution with a mean value of 0 and a standard deviation of 1. The equation is as follows:

(2) $$x^{\prime }=\frac{x-\bar{x}}{\sigma }$$

#### 2.3.2. Data Calculation Layer

**Figure 3:**
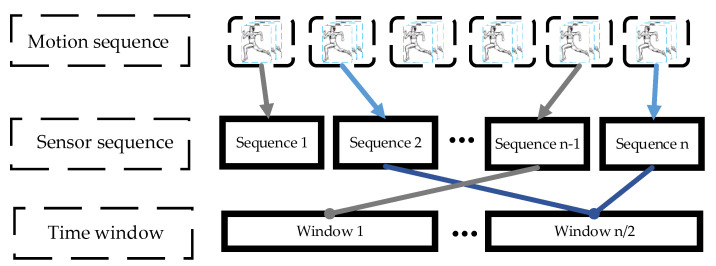
The moving time series data segmented using a sliding window.

The motion data, after preprocessing and recognition, are stored in the database in the data calculation layer. The preprocessed data is stored in the database in the form of a data stream, but it is difficult to obtain useful information from this type of data; as such, the frequency domain features of the data need to be extracted [28]. Sliding window is adopted to segment the time series data to produce a series of motion signal segments. The size of the window needs to be set when the sliding window is used, and the window should include a complete movement process. The size of the sliding window is set to twice the sampling frequency of the sensor to extract the complete motion features from the motion data. Figure 3 presents the process of dividing motion data by an active window.

The extraction of data features is the basis of identifying human movements. In the research process, the time-domain, frequency-domain, and time-frequency features of data are usually extracted as the data basis of feature recognition. Frequency analysis and time-domain analysis are adopted to extract data features.

**Figure 4:**
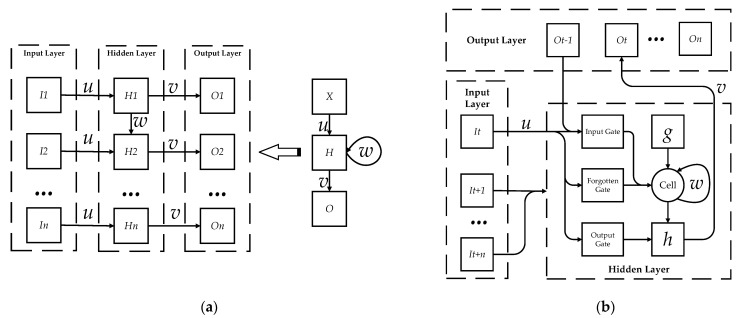
Comparison of neural network topological structure between RNN and LSTM: (**a**) the RNN neural network structure expanded according to the time series, and (**b**) the LSTM neural network topology diagram.

In the field of artificial intelligence, deep learning technology is widely used in facial recognition, driverless cars, and speech recognition. RNN is widely used in time series prediction and natural language recognition because it has the ability to mine time series data and correlate information before and after semantics. It takes the series data as the input value of the network, and realizes the recursion of the series data by chaining the cycle units; therefore, it is suitable for processing time series data. However, the gradient information of the RNN model will gradually disappear with an increase of time, so that the early feedback information retained in the late network operation is reduced. LSTM is an improvement of the RNN model. The “gate” structure in the LSTM network can selectively filter the input data information and extract valuable information from historical data [29]. The LSTM model is adopted to analyze the motion time series, and the human action is recognized according to the analysis results of the model. Figure 4 displays the comparison of neural network structure between RNN and LSTM.

Figure 4a shows the RNN structure expanded according to the time series. The left side is the network structure diagram of the node, and the right side is the schematic diagram of the node expanded according to the input time series sequence. Each node is composed of the input layer, hidden layer, and output layer, and the weights of each layer are *u*, *w*, and *v*, respectively. It suggests that some information will remain in neurons after each cycle when the data is transferred in RNN. It enters the next neuron as input and new information, and exerts an impact on the subsequent output data. At time *t*, the equations of the input vector $\alpha_{h}^{t}$ of the input layer, input vector $\beta_{h}^{t}$ of the hidden layer, and output vector $\gamma_{o}^{t}$ of the output layer are as follows:

(3) $$\alpha_{h}^{t}=\sum_{i=1}^{I}w_{ih}x_{i}^{t}+\sum_{h^{\prime }}^{H}w_{h^{\prime }h}b_{h^{\prime }}^{t-1}$$

(4) $$\beta_{h}^{t}=\theta_{h}(\alpha_{h}^{t})$$

(5) $$\gamma_{o}^{t}=\sum_{h=1}^{H}w_{ho}b_{h}^{t}$$

$w_{ih}$, $w_{h^{\prime }h}$ and $w_{ho}$, respectively, represent the weight parameters of the input layer, hidden layer, and output layer, and *I*, *H*, and *o*, respectively, represent the number of neurons in the input layer, hidden layer, and output layer; $\theta_{h}\left(\;\right)$ is the activation function of the hidden layer [30].

Figure 4b presents the topology of the LSTM neural network. Compared with RNN, the LSTM neural network propagates the hidden layer state *h_t_* and unit state *C_t_* forward in the process of information processing, and controls the state of the hidden layer at the previous time through the forget gate. The output value *f_t_* is determined by the state *h_t-1_* of the hidden layer at the previous time and the current input value *x_t_*. The equation is as follows:

(6) $$f_{t}=\sigma (w_{f}h_{t-1}+u_{f}x_{t}+b_{f})$$

**Figure 5:**
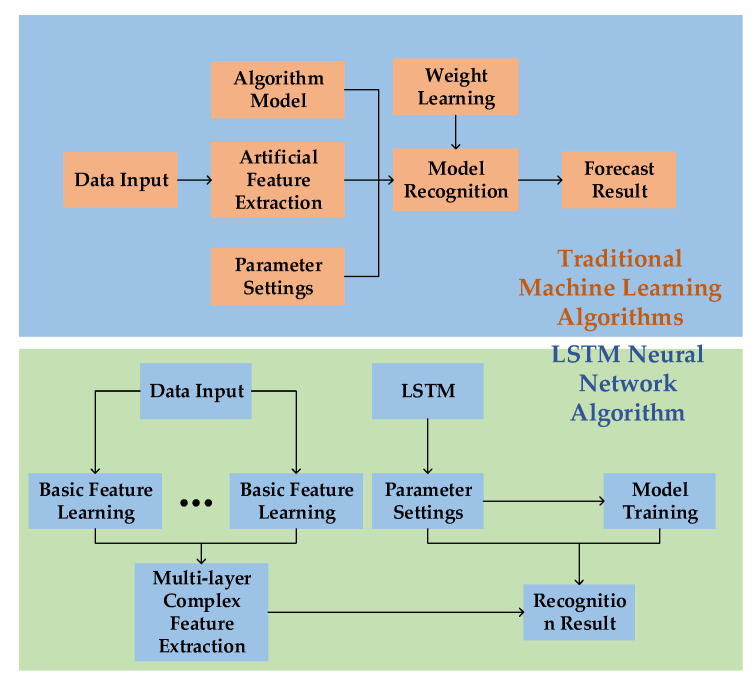
Comparison of data-processing flow between the LSTM neural network and traditional machine learning algorithms.

*w_f_*, *u_f_*, and *b_f_* represent the weight and bias matrix of the forget gate respectively; σ( ) represents the sigmoid function. Therefore, the LSTM neural network model can memorize historical data. The forget gate can be used to ignore useless motion data information, solve the long-term dependence problem of the RNN model, intelligently learn unlabeled data features, and effectively recognize human motion information [31]. In the process of motion information mining, the LSTM neural network can not only identify the relationship among motion sequence features, but also combine relatively simple motion features to form new complex features. It makes the weight replacement process more convenient and improves the efficiency and accuracy of human motion recognition [32]. Figure 5 presents a comparison of data processing between the LSTM neural network and traditional machine learning algorithms, and highlights the ability of LSTM to extract multi-layer complex features and avoid the problem of data information loss caused by data dimensionality reduction.

#### 2.3.3. Data Visualization Layer

The data visualization layer serves as the intermediary layer between the intelligent wearable device and the outside world. Teachers can access the system through a web browser and be presented with real-time movement data of students through the human–computer interaction interface. Developed web applications mainly use MVC architecture to deal with the relationship between interface, business logic, and data; reduce the cost of system maintenance through modular structure design; and improve the scalability of the system [33].

### 2.4. Database Introduction

The standard open dataset PAMAP2, proposed by the University of California, Irvine, was adopted as the training and testing database of human action recognition on Matlab R2014a for accuracy testing of the designed human action recognition algorithm. The total number of data is 3.85 million, including 52 attributes. The dataset includes acceleration and IMU (inertial measurement unit) data of different body parts from nine subjects wearing three inertial measurement devices and heart rate monitors in 18 daily activities such as walking, running, riding, and playing football. The sampling frequency of each sample is 100 Hz, and the total recording time of data is more than 10 h [34]. In order to verify the feasibility and effect of the designed human motion recognition algorithm for student motion recognition, the data was divided into the training set and test set in the ratio of 8:2. Then, the trained human action recognition model was adopted to recognize the action data in the testing set.

In order to better compare the classification effect of the classifier, the accuracy and confusion matrix are employed as the measurement standard to evaluate the classification effect of the classifier [35]. In the confusion matrix, *TP* means that positive samples are classified as positive samples; *FP* means that negative samples are classified as positive samples; *FN* means that positive samples are classified as negative samples; *TN* means that negative samples are classified as negative samples. The related evaluation indexes are presented below:

(1) *Accuracy* describes the classification accuracy of the classifier.

(7) $$\mathrm{Accuracy}=\frac{TP+FN}{TP+FP+TN+FN}$$

(2) *Precision* is the total proportion of positive samples correctly classified by the classifier.

(8) $$\mathrm{Precision}=\frac{TP}{TP+FP}$$

(3) *Recall* is the percentage of all positive samples that are correctly predicted.

(9) $$\mathrm{Recall}=\frac{TP}{TP+FN}$$

(4) *F*1 can be used to analyze the accuracy and recall of the model as a whole.

(10) $$F1=\frac{2(\mathrm{Precision}\times \mathrm{Recall})}{\mathrm{Precision}+\mathrm{Recall}}$$

## 3. Model Training and Testing

### 3.1. Training of LSTM Neural Network Model

**Figure 6:**
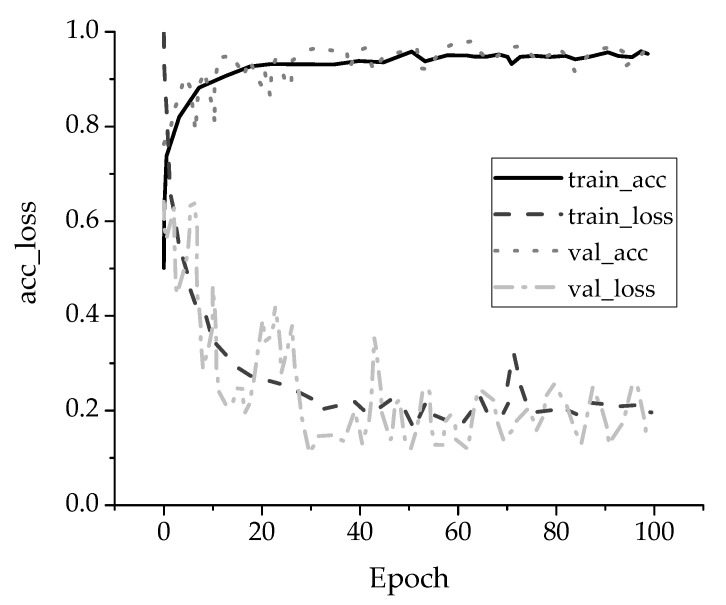
Training curve of acc and loss of LSTM neural network model.

Training is needed until the output results meet the accuracy of system recognition, so as to improve the accuracy of the model to recognize human body movement. The LSTM model can record movement characteristics and adjust the parameters of the model continuously during the training process. The training effect of the model is improved through repeated training. Figure 6 displays the change in accuracy (acc) and loss value (loss) of the LSTM neural network model over 100 iterations of training.

Figure 6 reveals that, in the iterative process, the accuracy of model recognition increases with the number of iterations, and the final recognition accuracy is close to 1. The loss value of the model gradually decreases with an increase in the number of iterations, but the overall value fluctuates and the final loss value of the model is close to 0.2. Therefore, the recognition accuracy and loss of the training model meet its needs. The model can be adopted to the identification of other motion categories through the storage and training of different motion feature data.

### 3.2. Model Test and Performance Comparison of LSTM Neural Network

**Figure 7:**
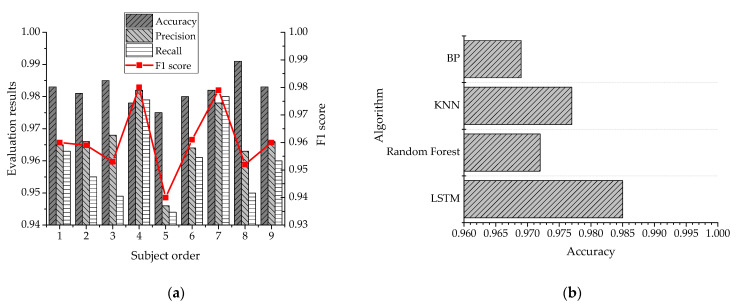
The results of the human action recognition model for different subjects: (**a**) the action recognition of the model for nine subjects in the testing set, and (**b**) the human action recognition accuracy of the LSTM neural network model compared with traditional algorithms.

In order to verify the recognition accuracy of the LSTM neural network, the model recognition results were compared with the real motion categories. The trained model was employed to recognize the data of nine subjects in the test set, and the recognition effect of the model was evaluated using accuracy, precision, recall, and F1 scores. The recognition accuracy of the LSTM algorithm was compared with random forest, k-nearest neighbor (KNN), and BP neural networks trained by the PAMAP2 dataset. Figure 7 displays the test results.

Figure 7a reveals that the trained human action recognition model has an accuracy of more than 97.5%, a precision of more than 94.6%, a recall rate of more than 94.4%, and an F1 score of more than 94.0% for the different subjects; the designed LSTM model has excellent human action recognition ability after training. In Figure 7b, the comparison of performance results with those of different algorithms also demonstrates that the designed model performs better than traditional algorithms—such as a neural networks—in addressing human action recognition problems. The recognition accuracy of 98.3% also verifies the strong recognition ability of the model. The reason for this may be that the LSTM neural network can capture the characteristics of time series data, optimize the data-processing process, and improve the accuracy of human motion recognition by adjusting the model parameters.

## 4. Discussion

The extraction of motion parameters such as motion recognition and posture judgment in the physical education classroom was shown to provide accurate feedback of student motions for teachers, and this recognition of student body movements can provide real-time feedback to improve the quality of training. Data from sensors in intelligent wearable devices were adopted to accurately identify human actions, but the controversial issue is the classification of patterns. The experimental results demonstrate that the human action recognition model based on the LSTM neural network can effectively recognize human action with an accuracy of more than 97.5%. In recent similar research, the recognition accuracy of a human motion recognition algorithm with six-stream CNN features of regional sequence established by Miao et al. (2018) was found to be 94.2% [36]. The recognition accuracy of an RNN-based VA-RNN and CNN-based VA-CNN network designed by Zhang et al. (2018) was 96%. None of these exceed the LSTM neural network accuracy of 97.5%. Thus, the model established here has the highest accuracy and the strongest recognition of those discussed [37,38].

With the development of human action recognition information technology and the application of deep learning, the human action pattern recognition algorithm based on wearable devices overcomes the limitations of traditional classification algorithms. It can effectively identify the pattern characteristics of student movement information in sports activities, and speeds up the research process of intelligent recognition of human movement patterns, as well as the new curriculum reform under the background of physical education curriculum development optimization [39,40].

Based on the analysis of results, it is concluded that the method proposed satisfied the expected research objectives, and valuable research conclusions can be drawn. A human motion recognition model based on LSTM was established, and the recognition accuracy of the training model is over 97.5%, which surpasses that of traditional human motion recognition algorithms. However, due to the limited academic level, some limitations exist. For example, the trained human recognition model proposed has a strong ability to recognize the moving images in the constructed test set. However, due to the complexity of the physical education environment, more interference information and other confounding factors are likely to exist. The proposed algorithm has yet to be verified in the physical education environment. Therefore, the motion recognition artificial intelligence technology for middle school student sports and the human body recognition ability of wearable devices proposed here need to be verified in subsequent studies. Follow-up work includes the collection of relevant data under different sports application scenarios, production of data sets, further training of the related algorithm models, and optimization of the algorithm.

## 5. Conclusions

The optimization of physical education teaching activities under the background of the new curriculum reform represents a crucial problem. Changes in the demands of physical education teaching under the new curriculum reform were analyzed and considered alongside the needs of social politics and the economy under the development of information technology [41]. It is proposed to apply artificial intelligence technology to physical education teaching activities. Artificial intelligence technology was adopted to establish a human action recognition model based on an LSTM neural network to identify the sports state of students in physical education teaching activities and to provide feedback on the physical condition of students to teachers so as to improve the quality of physical education activities. The results demonstrate that the designed human body recognition algorithm has good recognition accuracy which can provide timely and effective feedback on the students’ movement state in the physical education classroom. Under the background of the new curriculum reform, the proposed algorithm can meet the needs of the development of physical education in this new period, assist teachers to teach students according to their aptitude, significantly improve the physical quality of students, improve the traditional physical education classroom model, and protect students from potentially dangerous situations due to student-specific factors—thereby potentiating new ideas and means for physical education teaching under the new curriculum reform in China [42].

The original expected research objectives were fundamentally achieved, and valuable research conclusions were obtained. However, there exist some research deficiencies due to limited academic literacy. For example, the trained human body recognition model demonstrated a good recognition effect for the data in the dataset; however, factors in the actual environment are more complex, and the proposed algorithm has not been tested in the physical education teaching environment. Therefore, the human body recognition effect of wearable devices based on artificial intelligence technology needs to be verified and improved. Follow-up work will focus on collecting relevant data in different practical application scenarios, making datasets, and further training the relevant algorithm models.

## Figures and Tables

**Table 1:** Main functions of artificial intelligence wearable devices in sports activities.

Name	Equipment Function	Function of Artificial Intelligence Algorithm	Recognition Accuracy
Student physical condition detector	Monitoring the physical condition of students in real-time.	Neural network algorithm can recognize students’ human actions [19].	92.2%
	Improving the quality and performance of students’ physical training. In the training process, the sensors worn by students can accurately and timely detect movement.	The motion recognition algorithm is adopted to identify the motion parameters of the whole motion process, so as to effectively guide the students’ motion process [20].	86.4%
Marathon runner heartbeat monitor	Monitoring the heartbeat level and rhythm of athletes.	Intelligent devices are used to monitor and predict the heart rate of athletes and predict their heartbeat rhythm to prevent sudden death.	89.3%
Intelligent mouth guard	Monitoring oral status, molar status, dehydration, and concussion of athletes.	The algorithm identifies oral movements, detects and predicts the water content of athletes, and identifies the user’s travel status to detect whether unexpected conditions exist.	77.6%
Sweat analyzer	Analyzing human metabolism through the composition of sweat during competition and training.	Sweat components are analyzed to detect and predict the physical condition of athletes, and to provide corresponding medical and health care suggestions.	93.6%

## Data Availability

The raw data supporting the conclusions of this article will be made available by the authors, without undue reservation.
