# Expression of Concern: Liu et al. Optimization of a Sports Activity Development Model Using Artificial Intelligence Under New Curriculum Reform. *Int. J. Environ. Res. Public Health* 2021, *18*, 9049

**DOI:** 10.3390/ijerph23030396

**Published:** 2026-03-20

**Authors:** IJERPH Editorial Office

**Affiliations:** MDPI AG, St. Alban-Anlage 66, 4052 Basel, Switzerland; ijerph@mdpi.com

With this notice, the *IJERPH* Editorial Office alerts readers to concerns related to this article [1]. Following publication, concerns were raised regarding the reliability of certain references within the article, which may affect the scientific integrity of its content. In order to maintain the highest scientific standards, the Editorial Board and Editor-in-Chief have decided to issue this expression of concern while an investigation is ongoing.

The authors of this publication have been contacted for further clarification, but did not respond. 

Once this process has been completed, the Editorial Office will provide an update on the situation and announce any post-publication modifications deemed necessary, as per MDPI’s Update to Published Papers policy (https://www.mdpi.com/ethics#_bookmark30).
